# Comorbidities Associated with Large Abdominal Aortic Aneurysms

**DOI:** 10.1055/s-0039-1692456

**Published:** 2019-12-05

**Authors:** Verena Müller, Milena Miszczuk, Christian E. Althoff, Andrea Stroux, Andreas Greiner, Helena Kuivaniemi, Irene Hinterseher

**Affiliations:** 1 Surgical Clinic, Campus Charité Mitte and Campus Virchow-Klinikum, Charité – Universitätsmedizin Berlin, corporate member of Freie Universität Berlin, Humboldt-Universität zu Berlin, and Berlin Institute of Health, Berlin, Germany; 2 Vascular Surgery Clinic, Charité – Universitätsmedizin Berlin, corporate member of Freie Universität Berlin, Humboldt-Universität zu Berlin, and Berlin Institute of Health, Berlin, Germany; 3 Institute of Radiology, Campus Charité Mitte, Charité – Universitätsmedizin Berlin, corporate member of Freie Universität Berlin, Humboldt-Universität zu Berlin, and Berlin Institute of Health, Berlin, Germany; 4 Institute of Medical Biometrics and Clinical Epidemiology, Charité – Universitätsmedizin Berlin, corporate member of Freie Universität Berlin, Humboldt-Universität zu Berlin, and Berlin Institute of Health, Berlin, Germany; 5 Division of Molecular Biology and Human Genetics, Department of Biomedical Sciences, Faculty of Medicine and Health Sciences, Stellenbosch University, Tygerberg, South Africa

**Keywords:** abdominal aortic aneurysm, comorbidities, risk factor, lung function, smoking, diverticulosis

## Abstract

**Background** Abdominal aortic aneurysm has become increasingly important owing to demographic changes. Some other diseases, for example, cholecystolithiasis, chronic obstructive pulmonary disease, and hernias, seem to co-occur with abdominal aortic aneurysm. The aim of this retrospective analysis was to identify new comorbidities associated with abdominal aortic aneurysm.

**Methods** We compared 100 patients with abdominal aortic aneurysms and 100 control patients. Their preoperative computed tomographic scans were examined by two investigators independently, for the presence of hernias, diverticulosis, and cholecystolithiasis. Medical records were also reviewed. Statistical analysis was performed using univariate analysis and multiple logistic regression analysis.

**Results** The aneurysm group had a higher frequency of diverticulosis ( *p* = 0.008). There was no significant difference in the occurrence of hernia ( *p* = 0.073) or cholecystolithiasis ( *p* = 1.00). Aneurysm patients had a significantly higher American Society of Anesthesiology score (2.84 vs. 2.63; *p* = 0.015) and were more likely to have coronary artery disease ( *p* < 0.001), congestive heart failure ( *p* < 0.001), or chronic obstructive pulmonary disease ( *p* < 0.001). Aneurysm patients were more likely to be former ( *p* = 0.034) or current ( *p* = 0.006) smokers and had a significantly higher number of pack years ( *p* < 0.001). Aneurysm patients also had a significantly poorer lung function. In multivariate analysis, the following factors were associated with aneurysms: chronic obstructive pulmonary disease (odds ratio, OR = 12.24; *p* = 0.002), current smoking (OR = 4.14; *p* = 0.002), and coronary artery disease (OR = 2.60; *p* = 0.020).

**Conclusions** Our comprehensive analysis identified several comorbidities associated with abdominal aortic aneurysms. These results could help to recognize aneurysms earlier by targeting individuals with these comorbidities for screening.

## Introduction

Abdominal aortic aneurysm (AAA), a dilatation of the infrarenal aorta of ≥ 3 cm, 1 affects mostly older males. 1 The age group of 60 to 80-year-olds is growing, making AAA an increasingly important disease. AAA is often asymptomatic and diagnosed as an incidental abdominal imaging finding. A AAA screening program was introduced in the United States in 2007 2 and in Germany in 2016. 3

Smoking is the most important risk factor for AAA, increasing the risk for developing an AAA by 7.6-fold. 4 Other known risk factors include being male, age ≥ 65 years, family history for AAA, coronary artery disease (CAD), myocardial infarction, high blood pressure, and chronic obstructive pulmonary disease (COPD). 5 Protective factors include female sex, Type 2 diabetes, and a black African ethnicity. 1

Several diseases co-occur with AAA: cholecystolithiasis (CCL), 6 renal cysts, 7 COPD, 5 and hernias 8 9 are prevalent in the elderly population and share common risk factors with AAA, such as higher age and a positive smoking history. 7 10

Our goal was to identify additional diseases associated with AAA. They may have similar pathophysiology and could provide a better understanding of the disease, facilitating development of new therapies.

## Materials and Methods

The study was approved by the Charité Ethics Committee (approval number: EA1/309/16).

### Study Groups

Two study groups were created (100/group): (1) AAA group and (2) age- and sex-matched (±2 years) control group. All 200 individuals had undergone an abdominal computed tomography-angiography (CTA). AAA was defined as the infrarenal diameter of the aorta ≥ 3 cm. 11 The AAA patients were operated for AAA from 2004 to 2012 at the Surgical Clinic of Charité Mitte, Berlin, Germany (94 elective and 6 emergency operations). We used the patient's age when diagnosed with AAA, or if this information was missing, the age at the time of AAA operation.

The control patients had no history of AAA surgery, their infrarenal aortic diameter was < 3 cm, and had no other aneurysms. The control patients had received CTA scans at the Department of Radiology, Charité, Berlin, Germany, in 2005 to 2014 either for the evaluation of a kidney donation ( *n* = 14) or due to screening for melanoma recurrence ( *n* = 86). We used the age at the time of the CTA examination.

### Clinical Data

Details of the variables analyzed are described in the Data Dictionary ( Supplementary Table S1 ). The information was collected from the medical records and CTA images. We also reviewed findings from abdominal ultrasound examinations, the heart (ejection fraction), and lung function. As FEV1/VC (forced expiratory volume in 1 second in % of vital capacity) is the most reliable indicator of the exact status of the pulmonary function (restrictive versus obstructive disease), we chose this for the analysis.

The CTA images were evaluated using Centricity eRadCockpit (GE Healthcare, Chalfont St Giles, United Kingdom) for the presence of hernias, diverticulosis, CCL and previously performed cholecystectomy (CCE), and hiatal hernia by two investigators independently. Incisional hernias were excluded from the analysis, as all AAA patients underwent surgery and were exposed to the risk of incisional hernia.

The AAA diameter at initial diagnosis and surgery, graft material used during the operation, and family history were also recorded.

### Statistical Analyses

Analyses were performed using SPSS statistical package (v22 for Windows, IBM, Armonk, NY). First, a univariant comparison of the two groups was made. For quantitative variables, the mean, median, standard deviation, maximum and minimum values were determined. The categorical variables were described by means of cross tabulation. Differences between the two groups were assessed by Mann–Whitney U tests and chi-squared tests (Fisher's exact test). A *p* -value ≤ 0.05 was considered significant.

To identify the parameters independently associated with AAA, a multivariate analysis was performed and included multiple logistic regression models. Parameters with > 50% of values missing were not included in the analysis. ORs and 95% confidence intervals were calculated.

## Results

The two study groups included 100 AAA and 100 control patients (79 men and 21 women/group). The mean age in the AAA group was 69 ± 8.86 years, and in the control group 71 ± 8.59 years ( *p* = 0.065). The average AAA diameter at first contact was 51.78 ± 14.74 mm (median: 48 mm; range: 30–110 mm). The AAA diameter at the time of the surgery was 58.76 ± 12.36 mm (median: 57 mm). The smallest operated aneurysm was 34 mm in a patient whose rupture-prone aneurysms in the two common iliac arteries required immediate surgery and the small AAA was repaired during the same operation.

Only two (2%) patients, and none of the controls had a family history of AAA. One AAA patient had died prior to the AAA surgery, and two during hospitalization. Thus, the hospital mortality was 3%. One AAA patient was diagnosed with Marfan syndrome and was excluded from further analyses. There were no differences in the mean height, weight, or body mass index (BMI) between the study groups ( Supplementary Table S2 ).

AAA patients suffered more frequently from CAD ( *p* < 0.001) and had had a coronary bypass surgery more often ( *p* = 0.007), but not coronary stents ( *p* = 0.068) ( Table 1 ). Myocardial infarction ( *p* = 0.035) and congestive heart failure ( *p* < 0.001) were also more common in the AAA group. AAA patients were significantly more likely to have PAD ( *p* = 0.037). A comparison of the groups, using the Fontaine classification, showed no significant differences between the two groups ( *p* = 0.157).

**Table 1:** Clinical data gathered from medical records and computed tomography angiography images

Variables	AAA		Control		*p* -Value a
	With variable, *n*	Total with data, *n*	With variable, *n*	Total with data, *n*	
Hypertension	78	98	70	100	0.142
Coronary artery disease	49	98	19	100	< **0.001**
Coronary bypass	17	99	5	100	**0.007**
PTCA	23	99	13	100	0.068
Myocardial infarction	26	99	14	100	**0.035**
Peripheral artery disease	19	99	8	99	**0.037**
Peripheral artery disease stage: IIa/IIb/III/IV/bypass/PTA	5/7/2/2/3/0	99	2/1/1/1/2/1	99	0.157
Atrial fibrillation	14	99	10	100	0.497
Artificial pacemaker	2	99	1	100	0.497
TIA	6	99	3	100	0.323
Stroke	9	99	5	100	0.323
Congestive heart failure, all	20	99	2	99	< **0.001**
Congestive heart failure classes: NYHA 1/2/3/4	5/7/7/1	99	0/2/0/0	99	< **0.001**
Diabetes mellitus, all	18	99	19	97	0.856
Diabetes mellitus: diet/medication/insulin	10/6/2	99	0/14/5	97	**0.001**
Hyperlipidemia	35	98	34	98	1.000
COPD	28	98	4	100	< **0.001**
Ever-smoker	65	94	46	87	**0.034**
Current smoker	32	95	13	86	**0.006**
Hypothyroidism	10	99	6	100	0.311

Abbreviations: AAA, abdominal aortic aneurysm; COPD, chronic obstructive pulmonary disease; NYHA, New York Heart Association; PTA, percutaneous transluminal angioplasty; PTCA, percutaneous transluminal coronary angioplasty; TIA, transient ischemic attack.

a Chi-squared test.

Note: Refer Data Dictionary in Supplementary Table S1 for definitions of variables.

COPD was much more common in AAA patients ( *p* < 0.001). AAA patients were more likely to be former or current smokers. There was also a highly significant difference ( *p* < 0.001) in the mean and median number of pack years between the two groups (mean: 30.57, median: 25.00, range: 0–250 in the AAA group; mean: 10.89, median: 0, range: 0–60 in the control group). The ex-smokers from the control group had stopped smoking significantly earlier (mean: 14.54, median: 11.00, range: 0–45 years ago) than the AAA patients (mean: 5.63, median: 0.00, range: 0–50 years ago; *p* < 0.001).

The echocardiography and pulmonary function results demonstrated significant differences between the AAA and control groups ( Table 2 ).

**Table 2:** Ejection fraction and pulmonary function

Variables	AAA			Control			*p* -Value a
	Mean ± SD	Median	Data available, *n*	Mean ± SD	Median	Data available, n	
Ejection fraction (%)	54.04 ± 11.69	60	66 b	59.27 ± 6.02	60	26 c	0.075
FEV1 in % VC	66.85 ± 16.00	71	58 d	78.17 ± 8.82	80	21 e	**0.003**

Abbreviations: AAA, abdominal aortic aneurysm; FEV1, forced expiratory volume in one second; SD, standard deviation; VC, vital capacity.

a Mann–Whitney *U* test.

b Mean age 69 ± 8.99, 55 men, 11 women.

c Mean age 68 ± 10.08, 21 men, 5 women.

d Mean age 67 ± 8.55, 48 men, 10 women.

e Mean age 65 ± 10.98, 18 men, 3 women.

No significant difference was found in the frequency of CCL ( Table 3 ) or hernias ( Table 4 ) between the two groups. Diverticulosis was diagnosed significantly more often in the AAA group ( *p* = 0.006; Table 3 ). Five hiatal hernias were diagnosed in both groups ( *p* = 1.000). A splenic cyst was found in one AAA patient and three control patients ( *p* = 0.621). One AAA patient had a pancreatic cyst.

**Table 3:** Presence of cholecystolithiasis, diverticulosis, pancreatic, and splenic cysts based on computed tomography-angiography imaging data

Variables	AAA ( *n* = 99	Control ( *n* = 100)	*p* -Value a
CCL, ever	27	29	0.875
CCL, present	12	10	0.743
Cholecystectomy	15	19	0.743
Diverticulosis	44	31	**0.006**
Diverticulitis	4	0	**0.006**
Sigma resection	1	0	**0.006**
Pancreatic cysts	1	0	0.497
Splenic cysts	1	3	0.621

Abbreviations: AAA, abdominal aortic aneurysm; CCL, cholecystolithiasis.

Note: Computed tomography-angiography images were analyzed for 199 study patients. AAA patient with Marfan syndrome was excluded from the analysis.

a Chi-squared test.

**Table 4:** Presence of inguinal and umbilical hernia

	AAA ( *n* = 99)	Control ( *n* = 100)	*p* -Value a
Hernia, ever b	19 *c*	10	0.073
Inguinal hernia, ever	16	9	0.140
Inguinal hernia, present	5	4	0.280
Inguinal hernia, surgical repair	11	5	0.280
Umbilical hernia, ever	4	1	0.212

Abbreviations: AAA, abdominal aortic aneurysm.

Note: Computed tomography-angiography images were analyzed for 199 study patients. The AAA patient with Marfan syndrome was excluded from the analysis.

a Chi-squared test.

b All patients with a hernia diagnosis in their medical history.

c One patient had two types of hernia: inguinal and umbilical hernia.

The main differences in laboratory results were lower platelet count ( *p* = 0.035) and higher creatinine levels ( *p* = 0.009) in the AAA group ( Table 5 ). The AAA patients also had significantly higher thyroid-stimulating hormone levels ( *p* = 0.032). There were no differences in the ASA scores ( Supplementary Table S3 ), blood groups ( Supplementary Table S4 ), or Rhesus factor distributions ( Supplementary Table S5 ).

**Table 5:** Comparison of laboratory data between abdominal aortic aneurysm and control group

Variable	AAA			Control			*p* -Value
	Mean ± SD	Media, *n*	Data available, *n*	Mean ± SD	Median	Data available, *n*	
Hemoglobin	13.75 ± 1.95	13.85	98	13.29 ± 2.01	13.40	97	0.164
Hematocrit	0.41 ± 0.054	0.42	99	0.398 ± 0.054	0.41	97	0.188
Leukocytes	7.99 ± 3.09	7.23	99	8.67 ± 6.13	7.13	97	0.540
Platelet count	220.22 ± 58.41	211	99	253.51 ± 94.97	238	97	**0.035**
Glucose	115.12 ± 36.22	104	68	110.92 ± 43.56	96	51	0.092
CRP	14.21 ± 20.06	5.85	54	29.31 ± 54.14	3.85	76	0.455
Creatinine	1.33 ± 1.04	1.09	97	1.04 ± 0.39	0.93	97	**0.009**
TSH	2.12 ± 3.47	1.39	47	1.36 ± 1.22	1.13	75	**0.032**

Abbreviations: AAA, abdominal aortic aneurysm; CRP, C-reactive protein; SD, standard deviation; TSH, thyroid-stimulating hormone.

**Table 6:** Results of multivariate analysis after forward and backward selection

Parameter	OR	95% Confidence interval	*n*	*p* -Value
Ever smoker	1.488	0.371–5.960	123	0.575
PAD	0.979	0.566–1.693	123	0.938
Pack years	1.015	0.988–1.042	123	0.275
Congestive heart failure	2.408	0.352–16.468	123	0.370
ASA score	0.810	0.373–2,160	123	0.898
Diabetes mellitus	0.950	0.491–1.835	123	0.878
Coronary bypass	1.106	0.132–9.287	123	0.926
Creatinine	1.037	0.245–4.388	123	0.961
COPD	12.242	2.584–57.998	180	**0.002**
Current smoker	4.141	1.719–9.977	180	**0.002**
Coronary artery disease	2.603	1.160–5.838	180	**0.020**
Diverticulosis	1.844	0.941–3.615	180	0.075
Platelet count	0.994	0.989–0.999	180	**0.023**

Abbreviations: ASA, American Society of Anesthesiologists; COPD, chronic obstructive pulmonary disease; OR, odds ratio; PAD, peripheral arterial disease.

Results of the multivariate analysis showed the strongest association between COPD and AAA (OR = 12.242; *p* = 0.002). Current smoking (OR = 4.141; *p* = 0.002) and CAD (OR = 2.603; *p* = 0.020) also had a strong association with AAA ( Table 6 ). There was no direct association between AAA and the number of pack years (OR = 1.015; *p* = 0.275) or ever-smoking (OR = 1.488; *p* = 0.575). Diverticulosis showed a borderline association with AAA (OR = 1.844; *p* = 0.075). Platelet count revealed an indirect association with AAA (OR = 0.994; *p* = 0.023).

## Discussion

We found that COPD, CAD, congestive heart failure, and PAD were significantly more common among AAA patients than age- and sex-matched controls. The AAA patients received more coronary bypass implants, but not coronary stents. The control patients had a significantly higher FEV1/VC. Diverticulosis was observed significantly more frequently in AAA patients.

We identified COPD as the strongest independently associated disease with AAA, which is in agreement with previous studies reporting a prevalence of COPD up to 44% among AAA patients. 5 The FEV1 value is strongly associated with COPD. It is not surprising that the AAA patients had a significantly lower FEV1/VC ratio than controls (67 vs. 78%), a result comparable to other studies. 12 These findings are clinically important, especially for the surgical repair of AAA, since COPD is also a known risk factor for a poor outcome after a major surgery, 1 making it important to control COPD medically before a planned AAA surgery. Cronenwett et al 13 showed that the presence of COPD increases the risk of AAA rupture.

We found a strong association with AAA and cardiovascular diseases including CAD, congestive heart failure, and PAD. Some previous studies, 10 but not all, 14 showed an association between AAA and CAD. Several studies also mentioned an association between AAA and myocardial infarction, 15 which was confirmed in our study. Our study showed a significantly higher prevalence of congestive heart failure in the AAA group (20 vs. 2%), but no direct association with AAA in the multivariate analysis. One previous study found a prevalence of congestive heart failure to be 24% among AAA patients, 16 which is similar to our results.

Smoking is a well-established risk factor for AAA, 1 12 17 a finding confirmed in our study (OR = 4.141). The risk increases with the number of pack years, and even after stopping smoking, the increased risk remains apparent for decades. 17 Current smoking increases both the expansion and the rupture rate of AAA, as well as perioperative mortality. 17

Some studies, 8 but not all, 18 describe an association of hernias and AAA. Our study demonstrated higher prevalence of all hernias in the AAA (19%) than in the control group (10%); however, the difference was not significant. Alnassar et al 19 reported that 1.3% of AAA patients were diagnosed with an umbilical hernia, whereas in our study 4% of AAA and 1% of the control patients had an umbilical hernia. In both diseases, the inflammatory process is a prominent feature, 8 and mainly older men with a family history of smoking and COPD are affected. 9

AAA and CCL have rarely been examined together. We found a 12% prevalence of CCL among the AAA patients, which is slightly higher than most previous studies have reported (4.9–11%). 20 21 Schuster et al 6 revealed a significantly higher prevalence of CCL and CCE among AAA (50 and 11%, respectively) compared with controls (26 and 7%, respectively) and demonstrated an association between AAA and CCL in multivariate analysis. Our study could not confirm this finding.

In our study, 31% of control and 50% of AAA patients had a diagnosis of diverticulosis, but there was no direct association between diverticulosis and AAA in multivariable analyses. The prevalence of diverticulosis in the general population is 20 to 30% and rises sharply with older age. 22 Results of our study are, therefore, similar to the literature. Only one study has described an independent association between AAA and diverticulosis. 23 The Saint's Triad is thought to be a manifestation of a general connective tissue disorder, resulting in the formation of hernias, diverticula, aneurysms, or COPD. 23 If a diverticulitis develops, a diverticulum is more likely to perforate. In case of a free perforation, a peritonitis will occur and immediate colon surgery is necessary.

### Limitations

Limitations of our study are as follows: (1) It was a retrospective, single-center study of 100 AAA and 100 control patients. (2) Because the patient data were obtained from medical records and CT images, there is a possibility the data were incomplete. (3) The control group (kidney donation and melanoma patients) may not be representative of the general population. (4) We only compared operated AAA patients and the results presented here are, therefore, relevant for large AAAs.

Regarding the control group in our study, we would like to note that the findings agree with those from previous studies in which the control group consisted of patients who underwent thoracic and cardiovascular surgery 7 or had aortoiliac occlusive disease. 24 For example, Pitoulias et al 24 showed a significantly higher prevalence of COPD in the AAA study population as was seen in the current study. It could be argued that the melanoma patients formed an appropriate control group, since melanoma is an age-related disease and a disease of a different organ, not the aorta. There were no differences in the mean height, weight, or BMI between the AAA and control groups as shown in Supplementary Table S2 .

An advantage of our study was the matching of study groups for age and sex. This was all the more important because many of the diseases studied (e.g., CCL, diverticulosis, and COPD) are more common in the elderly population. Matching could also reduce the sex-related differences, since male sex is a known risk factor for AAA, while female sex is a risk factor for the CCL.

## Conclusions

This study provides a comprehensive characterization of the clinical picture of AAA and its associated comorbidities. The findings have several clinical implications emphasizing the need for a better risk stratification of AAA patients, which could lead to earlier detection of AAA. Screening programs for patients at risk could be created, and a cost–benefit analysis performed, especially among older men with a history of smoking. Other risk factors (e.g., COPD) should be taken into consideration. Our study confirmed that AAA patients often suffer from serious secondary diseases such as COPD, CAD, or heart failure. These are often the reasons for a poor postoperative outcome and increased mortality. Less serious diseases such as CCL and diverticulosis should not be forgotten. Comprehensive knowledge about AAA and its comorbidities facilitates future development of therapies.
