# Supplementary material for: Comorbidities Associated with Large Abdominal Aortic Aneurysms
Source: Aorta (Stamford). 2019 Dec 5;7(4):108–14. doi: 10.1055/s-0039-1692456 (PMC6914355; doi:10.1055/s-0039-1692456)
Supplement: Supplementary file 1 — Supplementary Material [file 10-1055-s-0039-1692456-s180019.pdf]

Supplementary Table S1 Data dictionary, in alphabetical order

Parameter	Definition	Obtained from
AAA diameter	At the time of initial diagnosis and surgery, in mm	CT
Age	AAA group: Age at initial diagnosis or, if missing, surgery<br>Control group: Age at CT scan	Medical records
Artificial pacemaker	ICD-10: Z95.0	Medical records
ASA score	ASA I—Normal, healthy patient<br>ASA II—Patient with mild systemic disease<br>ASA III—Patient with severe systemic disease<br>ASA IV—Patient with severe systemic disease that is a constant threat to life<br>ASA V—Moribund patient who is not expected to survive without the operation<br>ASA VI—Declared brain-dead patient whose organs are being removed for donor purposes	Medical records
Atrial fibrillation	ICD-10: I48.9	Medical records
Blood group	A/B/AB/0	Medical records
BMI	In kg/cm^2	Medical records
Cholecystolithiasis	Cholecystolithiasis ICD-10: K80.2<br>Cholecystectomy ICD-10: Z90.4	CT
Chronic kidney disease	Stage 1 ICD-10: N18.1<br>Stage 2 ICD-10: N18.2<br>Stage 3 ICD-10: N18.3<br>Stage 4 ICD-10: N18.4<br>Stage 5 ICD-10: N18.5	Medical records
Congestive heart failure	ICD-10: I50.0<br>NYHA class I/II/III/IV	Medical records
Connective tissue disease	Marfan syndrome ICD-10: Q87.4<br>Ehlers–Danlos syndrome ICD-10: Q79.6<br>Loeys–Dietz syndrome ICD-10: Q25.4	Medical records
COPD	ICD-10: J44.90–93	Medical records
Coronary artery disease	ICD-10: I25.1	Medical records
Coronary bypass	ICD-10: Z95.1	Medical records
Diabetes	ICD-10: E11.90<br>Treatment with:<br>Diet<br>Medication<br>Insulin	Medical records
Diverticulosis	Diverticulosis ICD-10: K57.0<br>Diverticulitis ICD-10: K57.12<br>Sigma resection ICD-10: Z90.4	CT
Ejection fraction	In %	Medical records
Family history of AAA	Does patient have any first or second degree relatives with AAA	Medical records
Height	In centimeters	Medical records
Hernia	Present or status post-surgery<br>Inguinal hernia ICD-10: K40.0<br>Umbilical hernia ICD-10: K42.0	CT
Hyperlipidemia	ICD-10: E78.9	Medical records
Hiatal hernia	ICD-10: K44.9	CT
Hypertension	ICD-10: I10.00 or medication	Medical records
Hypothyroidism	ICD-10: E03.9	Medical records
Liver cysts	ICD-10: K76.8<br>Size	CT

Supplementary Table S1 (*Continued*)

Parameter	Definition	Obtained from
	≤ 1 cm<br>> 1–≤3 cm<br>> 3–≤5 cm<br>> 5 cm	
Lung function (FEV1/VC)	In %	Medical records
Myocardial infarction	ICD-10: I25.2	Medical records
Nephrolithiasis	ICD-10: N20.0	CT
Other aneurysm	Presence of aneurysms in other arteries	CT
Pancreatic cysts	ICD-10: K86.2	CT
Peripheral artery disease	ICD-10: I70.29<br>Fontaine Stage I/IIa/IIb/III/IV<br>Bypass ICD-10: Z95.88<br>PTA ICD-10: Z95.88	Medical records
PTCA	ICD-10: Z95.88	Medical records
Rhesus factor	Rh +/Rh -	Medical records
Smoking	Current or former smoking ICD-10: F17.2<br>Years and amount of smoking (pack years)<br>Years since stopped smoking	Medical records
Splenic cysts	ICD-10: D73.4	CT
Stroke	TIA ICD-10: G45.99<br>Stroke ICD-10: I64	Medical records
Surgical material used	Graft material used in AAA surgery	Medical records
Urgency of AAA surgery	Elective, urgent, emergent	Medical records
Weight	In kilograms	Medical records

Abbreviations: AAA, abdominal aortic aneurysm; ASA score, American Society of Anesthesiologists; BMI, body mass index; COPD, chronic obstructive pulmonary disease; CT, computed tomography; FEV1/VC, forced expiratory volume in 1 second in % of vital capacity; ICD-10, International Classification of Diseases; NYHA, New York Heart Association; PTA, percutaneous transluminal angioplasty; PTCA, percutaneous transluminal coronary angioplasty; TIA, transient ischemic attack.

Supplementary Table S2 Comparison of height, weight, and body mass index (BMI) between the abdominal aortic aneurysm (AAA) and control group

Variable	AAA ($n = 99$)		Control ($n = 98$)		*p*-Value[a]
	**Mean ± SD**	**Median**	**Mean ± SD**	**Median**	
Height, cm	173.65 ± 9.18	174	174.01 ± 8.05	175	0.692
Weight, kg	80.50 ± 15.79	80	79.48 ± 13.99	76.50	0.355
BMI, kg/m^2	26.66 ± 4.68	26.53	26.18 ± 3.66	25.59	0.377

Abbreviations: AAA, abdominal aortic aneurysm; BMI, body mass index; SD, standard deviation.
Data were not available on two controls. Also, the AAA patient with Marfan syndrome was excluded from the analysis.
[a]Mann–Whitney *U* test.

Supplementary Table S3 ASA (American Society of Anesthesiologists) scores

Study group	ASA I	ASA II	ASA III	ASA IV	ASA V	Data available on, *n*	Average ASA score
**AAA group**	1	22	70	4	2	99	2.84 ± 0.601
**Control group**	3	34	61	1	1	100	2.63 ± 0.614

Abbreviations: AAA, abdominal aortic aneurysm; ASA, American Society of Anesthesiologists.

Supplementary Table S4 AB0 blood groups

Study group	A	B	AB	0	Data available on, *n*	*p*-Value chi-squared test
AAA group	41	16	6	34	97	0.964
Control group	31	12	3	23	69	

Abbreviation: AAA, abdominal aortic aneurysm.

Supplementary Table S5 Rhesus factor

Study group	Positive, *n*	Negative, *n*	Data available on, *n*	*p*-Value chi-squared test
AAA group	82	15	98	0.832
Control group	57	12	69	

Abbreviation: AAA, abdominal aortic aneurysm.
